# Modulation of Affective and Sensory Qualities of Acute Nociceptive Pain by Curcuma longa and Boswellia serrata Extract Formulation: A Randomized, Double-Blind, Placebo-Controlled Design in Subjects With Exercise-Induced Acute Musculoskeletal Pain

**DOI:** 10.7759/cureus.77204

**Published:** 2025-01-09

**Authors:** Sanjeev Kumar Kare, Girish H R, Ajay Gupta

**Affiliations:** 1 Orthopedics, Government Medical College and General Hospital, Srikakulam, IND; 2 Orthopedics, Rajalakshmi Hospital, Bangalore, IND; 3 Orthopedics, Nirmal Hospital, Jhansi, IND

**Keywords:** acute joint pain, antiinflammatory, boswellia serrata, : musculoskeletal pain, turmeric powder

## Abstract

Introduction

Excessive repetitive physical activity most often leads to acute musculoskeletal pain. The management of acute pain is one of the primary concerns. The nociceptive pain has both sensory and affective qualities, patterns, and intensity. In this article, we focus on the effect of a turmeric-*Boswellia* formulation on that aspect at different locations of the body.

Methods

This multicentric study with a randomized, double-blind, placebo-controlled design enrolled 232 subjects in a male-to-female ratio of 1:1. The test dosage was 1,000 mg of turmeric-*Boswellia* extract (TBE) and a similar placebo in a parallel design allocation of 1:1 ratio. Healthy subjects with acute musculoskeletal pain of exercise-related origin with a numerical pain rating score of ≥ 5 were included in the study. The study duration was six hours, and the efficacy was analyzed using the short form of the McGill Pain Questionnaire, having subscales visual analog scale (VAS) and present pain index (PPI).

Results

There was a significant reduction in pain in the McGill total score (p < 0.001) of the TBE group, with a 98% change from the baseline compared to the placebo. The sensory and affective domains showed a significant reduction of 98% (p < 0.001) and 97% (p < 0.001) in pain in the TBE group from the baseline compared to placebo. The VAS had a 97% (p < 0.001), and PPI had 96% (p < 0.001) pain relief in the TBE group from the baseline compared to placebo. In the sensory domain, the most common pain descriptor reported by the participant was “tender,” and in the affective domain, it was “tiring-exhaustive.” The descriptor frequency of “tender” reduced from 65% in baseline to 1%, and “tiring-exhaustive” reduced from 61% to 3% at the end of six hours for the TBE group, while the placebo showed negligible change. The mean pain intensity of all pain descriptors in the TBE group showed more than 95% change from baseline.

Conclusion

It can be concluded that the turmeric-*Boswellia* formulation was very effective for exercise-induced musculoskeletal pain irrespective of location and had a significant reduction in pain intensity for sensory as well as affective pain sensations.

## Introduction

Exercise-induced acute musculoskeletal pain (MSP) is usually caused by an acute bout of physical activity. Acute MSP affects various structures of the body, like muscles, bones, joints, ligaments, and tendons. The commonly affected areas are the upper and lower limbs, back, and neck, but nonlocalized widespread myalgia is also prevalent [1].

The musculoskeletal system is a common area of nociceptive pain experience. Nociceptors are pain receptors that detect something that can cause damage to the body, like physical force to muscles, bones or connective tissues, hot or cold temperatures, etc. Nociceptive pain is different from neuropathic pain as nociceptive pain is sensed in response to an external stimulus to the body, but neuropathic pain arises from damage to nerves and nervous systems. One can feel neuropathic pain in the limb even when there is no limb, called phantom limb syndrome.

Traditional pain measurement methods consider pain as a unidimensional quantity that varies only in intensity, like the verbal rating scale (VRS), visual analog scale (VAS), and the numerical rating scale (NRS) [2]. Although they provide simple and effective measures of pain intensity, their main disadvantage is the assumption that pain is a unidimensional experience. The experience of pain is much varied, not limited to a sensory dimension that the pain intensity scales measure but also extends to the psychologic (affective) and cognitive dimensions. The pain of a pinprick is different from a ligament sprain or strain, and the quality of pain differs in each case. Chang et al. reported that capsaicin injection into skin and muscle produced distinctly different pain qualities [3].

Investigators have long been aware of the different types of pain sensations and how difficult it is for someone who is experiencing pain to explain it to them. Descriptions of pain after conditions like peripheral nerve injury, such as burning, shooting, stabbing, or cramping qualities of visceral pain, provide key diagnosis and may even decide on the course of therapy. It is crucial to assess pain if research on how people experience it is to be reliable. If a medication's efficacy is in question, then we need figures to demonstrate a pain reduction. In addition to determining whether our medication precisely reduced the stabbing or burning character of the pain, we also need to ascertain whether the terrible or excruciating pain was also lessened.

Melzack and Torgerson, in 1971, developed a questionnaire called the McGill Pain Questionnaire (MPQ) [4]. The 20 pain descriptors were grouped into sensory, affective, evaluative, and miscellaneous. It also contained marking pain locations, pain rating index, and present pain index (PPI). Dubuisson and Melzack (1976) studied the discriminative capacity of the questionnaire by administering the questionnaire to patients with eight different pain syndromes: post-herpetic neuralgia, phantom limb pain, metastatic carcinoma, toothache, degenerative disc disease, rheumatoid arthritis or osteoarthritis, labor pain, and menstrual pain. Discriminant analysis revealed that each type of pain had a distinctive constellation of verbal descriptors [5,6]. Descriptor patterns can provide the basis for differentiating between two major types of low back pain. Leavitt and Garron (1980) [7] found that patients whose pain has no detectable cause (functional) use distinctly different patterns of words than those with physical ("organic") causes. The MPQ categorization based on the patients' chosen word patterns had an 87% concordance with the recognized medical diagnosis [5]. Roche et al. (2003) in his study reported that gnawing, heavy, aching, annoying, and exhausting were the words used by patients with rheumatoid arthritis [8].

In 1987, the short form of the MPQ was developed to be used in the research setting where time is constrained. The short form contained 15 pain descriptors with 11 words in sensory and four in affective, with each descriptor ranked on an intensity scale of 0 to 3 (none to severe). It had a VAS and PPI to provide indices of overall pain intensity. The descriptors were selected from the long form based on the frequency of endorsement by patients with a variety of acute, intermittent, and chronic pains. The short form has demonstrated good psychometric properties across the adult lifespan. Importantly, the same MPQ and SF-MPQ descriptors are chosen most frequently by different age groups to describe the same type of pain (e.g., arthritis pain), thus supporting the scales" construct and discriminative validity in recognizing the type of pain [9].

Management of pain is the central focus in clinics that deal with MSP, and knowledge of the quality of pain would better enable to address the concerns of the subjects. Turmeric and *Boswellia* are two herbal extracts that are well-studied for their analgesic and anti-inflammatory properties. A formulation of turmeric and *Boswellia* dispersed in sesame oil (Rhuleave-K, Arjuna Natural Pvt. Ltd., Aluva, India) has been reported to have fast pain relief in an open-label, single-center, acute MSP study [10]. Since it was an open-label study, a more rigorous double-blinded placebo-controlled multicenter study was conducted to substantiate the results previously obtained. This article discusses the management of sensory and affective aspects of nociceptive pain resulting from exercise-induced acute MSP, which was measured using short-form McGill Pain Questionnaire (SF-MPQ).

## Materials and methods

The study recruited 232 subjects with a male-to-female ratio of 1:1 for the whole study. The protocol for this randomized, placebo-controlled, multicenter study was approved by the respective institutional ethics committees of the study centers. The study was registered in the clinical trial registry of India (CTRI/2020/06/025601). This multicentric clinical trial was conducted across six institutes in India, with the trial periods varying by site. The overall study spanned from August 2020 to October 2020. The trial was conducted at Rajalakshmi Hospital, Bengaluru (05 August 2020 to 25 September 2020), Vagus Super Specialty Hospital, Bengaluru (25 August 2020 to 05 October 2020), Santosh Hospital, Bengaluru (18 August 2020 to 05 October 2020), GMC & GGH, Srikakulam (18 September 2020 to 29 September 2020), Nirmal Hospital, Jhansi (17 September 2020 to 03 October 2020), and Sudbhawana Hospital, Varanasi (18 September 2020 to 21 October 2020).

The participants were selected from the outpatient departments of hospitals of the respective sites. Participants aged 18-65 with acute exercise-induced MSP presenting at the site within 24 hours of occurrence who were otherwise healthy were selected for screening procedures. The participants gave voluntary written informed consent forms before participating in the screening procedures. The principal/co-investigator explained all the aspects of the study in detail.

Participants with an NRS score of 5 or greater were included in the study. Arthritis or osteoarthritis, acute muscle spasms requiring parenteral therapy or surgery, Grade 2 and 3 sprain or strain, and hospital admission for management of painful acute soft tissue injury of the upper or lower extremity, including acute injuries of ligaments and tendons were excluded. The study was done in accordance with the International Council for Harmonisation of Technical Requirements for Pharmaceuticals for Human Use (ICH) Guideline for Good Clinical Practice (GCP) and the Declaration of Helsinki.

A proprietary formulation of *Curcuma longa* and *Boswellia serrata* extract in black sesame seed oil (turmeric-*Boswellia* extract (TBE)) was used as the test product in the study. TBE was standardized to contain not less than (NLT) 26.6% curcuminoids and NLT 1% acetyl keto-boswellic acid (Rhuleave-K). The test product was reddish-brown colored vegetable soft gel capsules of 500 mg, and two capsules were administered as a single dose. A similar colored matching placebo was administered at the same dosage (2 × 500 mg).

The master randomization list was generated by an independent statistician using the software WinPepi version 11.65 (2016) (Joseph H Abramson, Jerusalem, Israel) using balanced stratified randomization in a 1:1 ratio. The random list was allocation-concealed using alphanumeric codes, and the intervention products (IPs) were packed in opaque bottles. The TBE and placebo were blinded using similar size, color, packaging, and labeling. The IPs were identified only by their allocation codes. Opaque and sealed envelopes with package inserts of the identity of each IP were kept under the custody of the pharmacist for unblinding purposes. In case of an emergency or a need to know the identity of the blinded IP, the investigator would request the pharmacist to provide the envelope of the required IP and promptly inform the sponsor of the need to do so.

In the study, reported pain was categorized according to the anatomical regions and segregated into five categories.

· General MSP

· Head and neck: neck

· Upper limb: shoulder, hand, arm, forearm, clavicle, wrist, and elbow

· Trunk: back and pelvic

· Lower limb: hip and thigh muscles, leg, foot muscles, knees, and ankle

The SF-MPQ primarily consisted of two major classes of word descriptors that are used by subjects to specify subjective pain experience: words that describe the sensory qualities of the experience in terms of temporal, spatial, pressure, thermal, and other properties and words that describe affective qualities in terms of tension, fear. The SF-MPQ consists of 15 descriptors of pain, 11 from the sensory and four from the affective categories of the MPQ. The words have pain intensity scoring with none = 0, mild = 1, moderate = 2, and severe = 3. The words were selected on the basis of their frequency of endorsement by subjects with a variety of acute, intermittent, and chronic pains [11]. The MPQ also contains PPI and VAS as subscales.

The McGill score for pain at each location was analyzed as sensory score, affective score, total score, PPI, and VAS score. The pain descriptors grouped under the affective score contain from “throbbing” to “splitting,” and the sensory score contains from “tiring-exhaustive” to “punishing-cruel.” All subjects were taken for analysis. The pools of data were analyzed using the NCSS2020 Statistic Program for Windows. The descriptive statistics of baseline characteristics were represented as mean, standard deviation, standard error, and minimum and maximum values. The analysis of pain location categories of MSP was done using the Mann-Whitney U test, t-test, or Wilcoxon signed-rank test.

## Results

The study enrolled 232 subjects, with 116 males and females. The average age in the TBE group was 37.18 years (n = 116), and that in the placebo group was 37.55 years (n = 116). There was no significant difference between the baseline parameters of age, height, and weight (p > 0.05 for all) for the two groups (Table 1).

**Table 1:** Baseline demographics of subjects in the placebo and TBE groups SD - standard deviation; SE - standard error; TBE - turmeric-*Boswellia* extract The between-group analysis was conducted using an independent sample t-test.

	Placebo (n = 116)						TBE (n = 116)						
	N	Mean	SD	SE	Min	Max	N	Mean	SD	SE	Min	Max	p-value
Age (years)	116	37.55	13.47	1.25	18	64	116	37.18	12.81	1.19	18	63	0.919
Height (cm)	116	166.69	8.67	0.81	148	182	116	166.85	9.34	0.87	148	185	0.747
Weight (kg)	116	69.75	11.59	1.08	43	95	116	69.81	11.55	1.07	45	95	0.846

**Figure 1:**
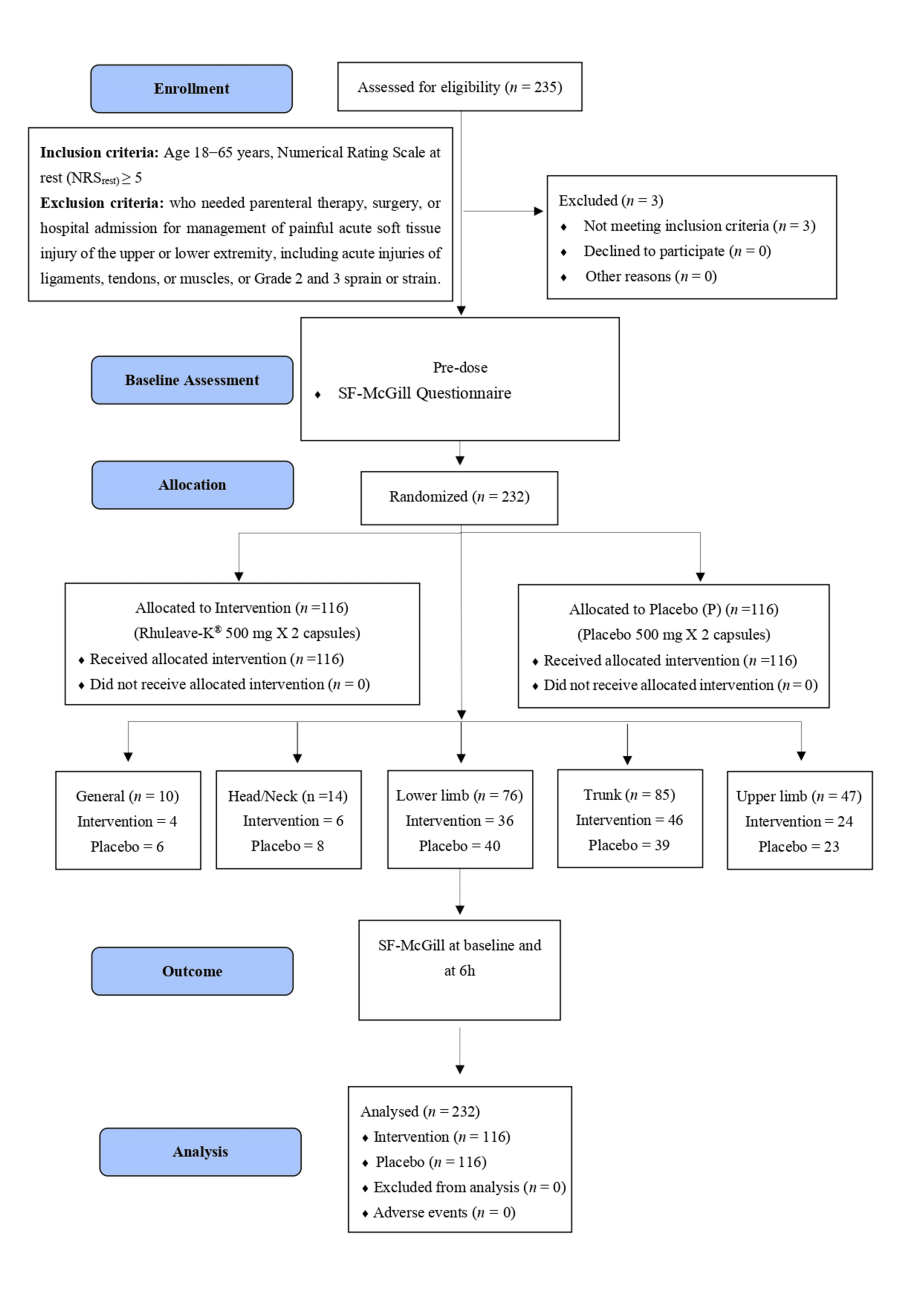
Participants flow diagram.

The study flow diagram is presented in Figure 1.

**Figure 2:**
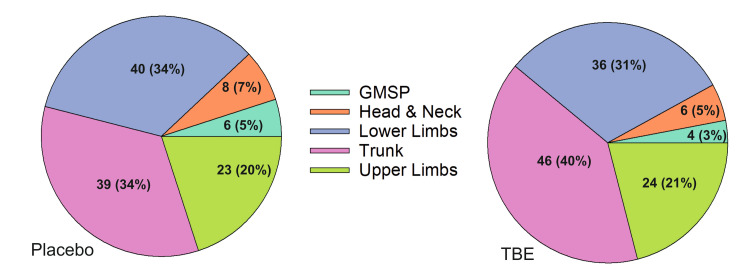
Distribution of exercise-induced musculoskeletal pain reported according to location of pain at baseline. The data are represented as n(%). Total number of participants in placebo and TBE, n = 116 per group.

The most common location of exercise-induced acute MSP presented by the participants were the trunk (34% in placebo and 40% in TBE) and lower limbs (34% in placebo and 31% in TBE), followed by upper limbs (20% in placebo and 21% in TBE) (Figure 2).

**Figure 3:**
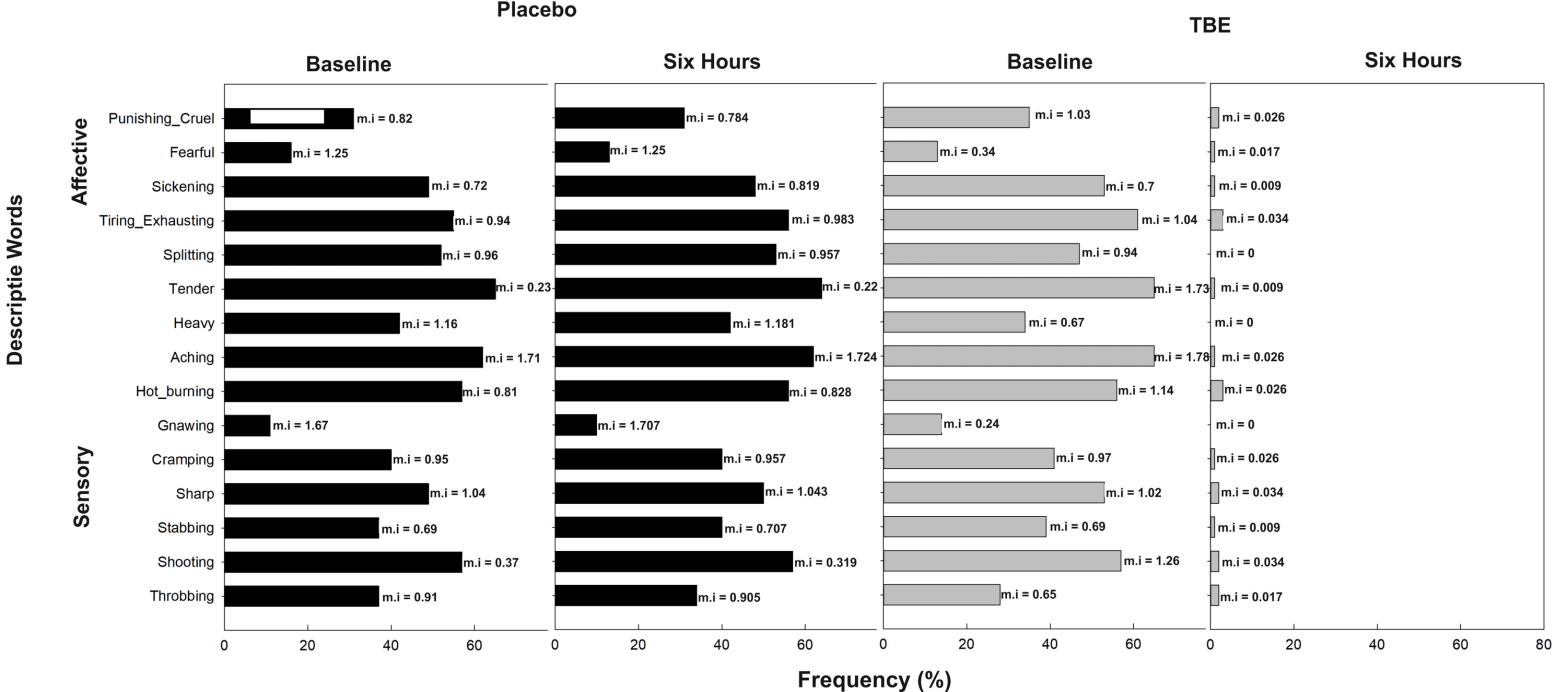
Frequency of subjects describing exercise-induced musculoskeletal pain (total) and their corresponding mean pain intensity (mi) using short-form McGill Pain Questionnaire at baseline and at the end of the study after six hours for placebo and turmeric-Boswellia extract (TBE). Mean pain intensity values, shown next to each bar, indicate the mean severity rating assigned by participants. Higher mi values reflect the greater perceived intensity of the described sensation or effect.

In the placebo group (n = 116), the most common descriptive word for pain was “tender” (65% mean pain intensity (mi) =1.67) and “aching” (62% mi =1.71) followed by “hot burning” (57% mi = 1.16), “shooting” (57%, mi = 1.25), and “splitting” (52% mi = 0.95) in the sensory dimension and “tiring” (55% mi = 1.04) in the affective followed by “sickening” (49% mi = 0.69). In the TBE group (n = 116), the most common descriptive words were also “aching” (65%, mi = 1.78) and “tender” (65%, mi = 1.73) followed by “shooting” (57%, mi = 1.26), “hot burning” (56%, mi = 1.14), “sharp” (53%, mi = 1.02) in the sensory and “tiring-exhaustive” (61%, mi = 1.04) in the affective followed by “sickening” (53%, mi = 0.70) (Figure 3).

**Figure 4:**
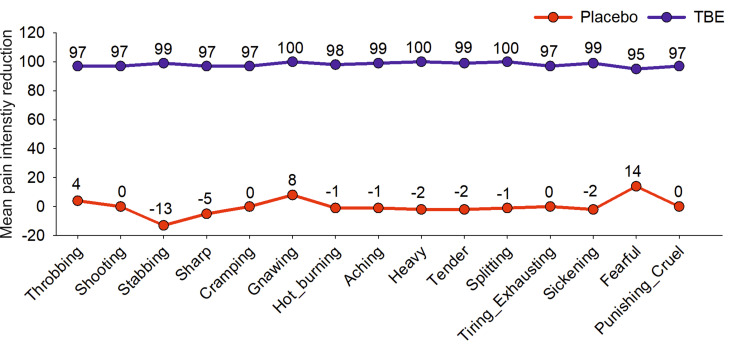
Mean pain intensity reduction (total) for each of the descriptor words at the end of six hours of study. TBE - turmeric-*Boswellia* extract

Post-dose after six hours in the TBE group, the subjects choosing the words “tender” and “aching” reduced to 1% with 99% reduction in mi, “shooting” and “sharp” frequency reduced to 2% with 97% reduction in mi, “hot burning” to 3% with 98% reduction in mi in the sensory domain and “tiring-exhaustive” to 3% with 97% reduction in mi, “sickening” to 1% with mi reduction of 99%. At the same time, the placebo had negligible change, often showing a slight increase in the mi of chosen words (Figure 4).

A Mann-Whitney analysis of the total sensory and affective domains between the two groups showed that there was a significant difference between the two groups (p < 0.001) in both sensory and affective dimensions, with 98% and 97% change, respectively.

**Figure 5:**
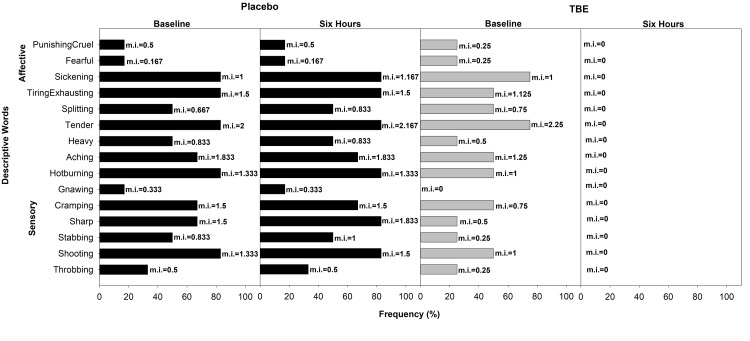
Frequency of subjects describing general musculoskeletal pain (GMSP) and their corresponding mean pain intensity (mi) using short-form McGill Pain Questionnaire at baseline and at the end of the study after six hours for placebo (n = 6) and turmeric-Boswellia extract (TBE) (n = 4). Mean pain intensity values, shown next to each bar, indicate the mean severity rating assigned by participants. Higher mi values reflect the greater perceived intensity of the described sensation or effect.

In the placebo group, at baseline, the most commonly used words to describe pain in the anatomical location-wise category of general MSP were “shooting” (mi = 1.33), hot-burning (mi = 1.33), and tender (mi = 2) in the sensory domain and tiring-exhaustive (mi = 1.5) in the affective domain with 83% frequency for all. Whereas in the TBE group, at baseline, “tender” (mi = 2.25) was the most common word used with 75% frequency, and 50% frequency for both “shooting” (mi = 1) and “hot-burning” (mi = 1) in the sensory domain and in the affective domain, the common words were “sickening” (75%, mi = 1) and “tiring-exhaustive” (50%, mi = 1.25). After the study of six hours, in the TBE group, there was a 100% reduction in the “shooting,” “hot-burning,” and “tenderness” aspects of pain in the sensory domain and “tiring-exhaustive” and “sickening” aspects of pain while in the placebo group “shooting” mi increased by 13%, “tender” by 8%, “sickening” by 17% with no change in the “hot-burning” and “tiring-exhaustive” aspects (Figure 5). Mann-Whitney analysis of the sensory (p = 0.03) and affective (p < 0.001) domains indicated that there was a significant reduction in the quality of pain in the TBE group.

**Figure 6:**
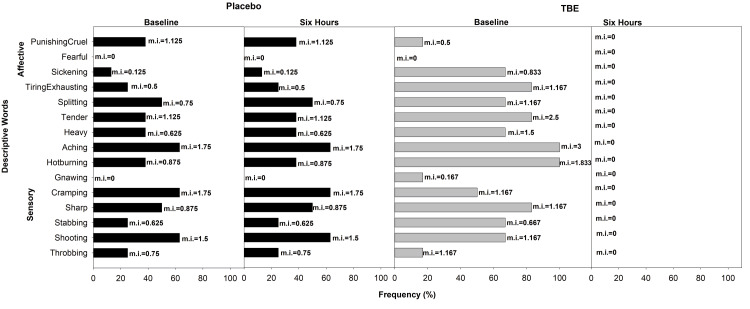
Frequency of subjects describing head and neck pain and their corresponding mean pain intensity (mi) using short-form McGill Pain Questionnaire at baseline and at the end of the study after six hours for placebo (n = 8) and turmeric-Boswellia extract (TBE) (n = 6). Mean pain intensity values, shown next to each bar, indicate the mean severity rating assigned by participants. Higher mi values reflect the greater perceived intensity of the described sensation or effect.

In the head and neck category of the placebo group, the most common word at baseline was “shooting” (mi = 1.5), “cramping” (mi=1.75), and “aching” (mi = 1.75), with 63% frequency in the sensory dimension and “punishing” in the affective dimension (38%, mi = 1.13). Whereas, in the TBE group, the highest was for “hot-burning” (mi = 1.83) and “aching” (mi = 3), with 100% in the sensory domain and “tiring-exhausting” (83%, mi = 1.17) in the affective domain. After the study of six hours, in the TBE group, there was a 100% reduction in all the pain descriptions, whereas in the placebo, there was no change (Figure 6).

**Figure 7:**
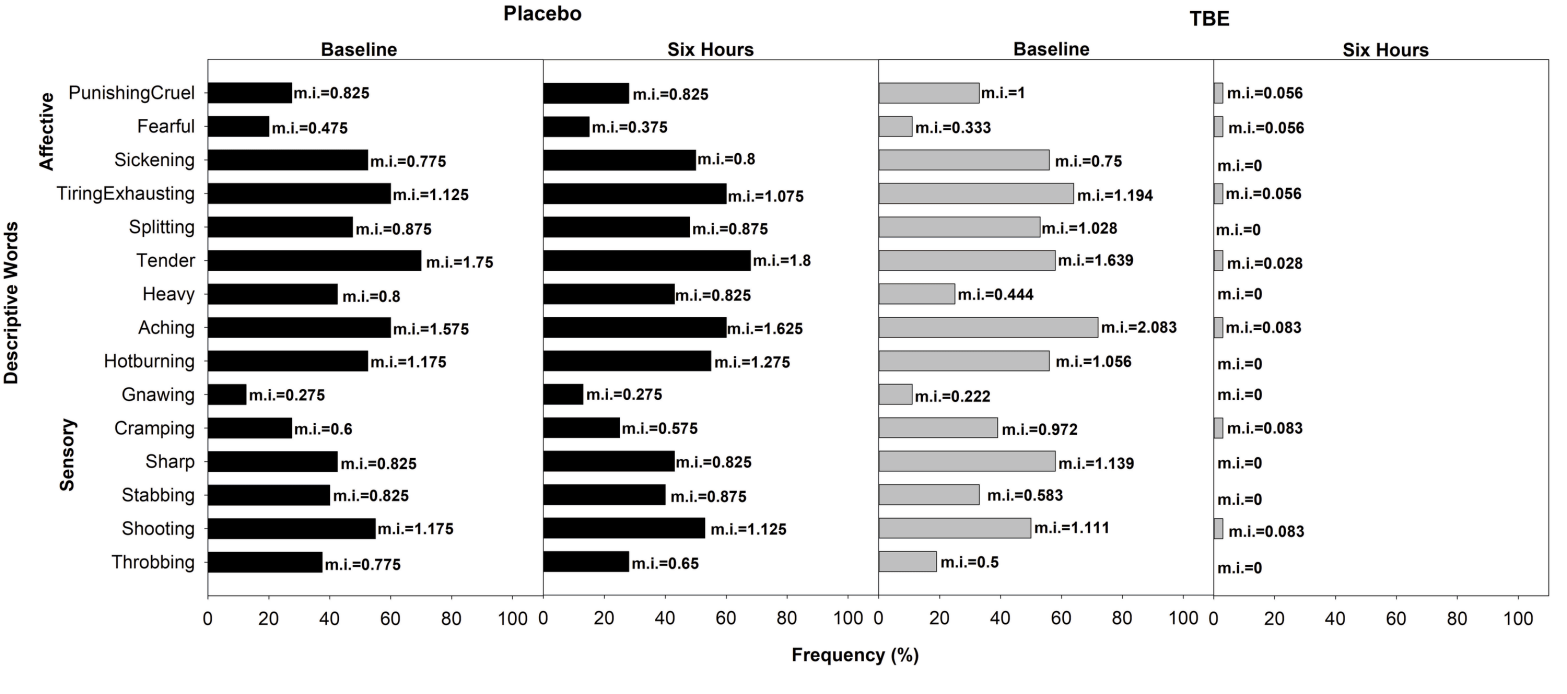
Frequency of subjects describing lower limb pain and their corresponding mean pain intensity (mi) using short-form McGill Pain Questionnaire at baseline and at the end of the study after six hours for placebo (n = 40) and turmeric-Boswellia extract (TBE) (n = 36). Mean pain intensity values, shown next to each bar, indicate the mean severity rating assigned by participants. Higher mi values reflect the greater perceived intensity of the described sensation or effect.

In the lower limbs category of the placebo group, the most common word was “tender” (70%, mi = 1.75) in the sensory group and “tiring-exhausting” in the affective domain (60%, mi = 1.125). In the TBE group, “aching” (72%, mi = 2.08) and “tiring-exhausting” (64%, mi = 1.19) were the common words used. After the study of 6 hours, in the TBE group, there was a 96% reduction in “aching” and 100% in “tiring-exhausting,” whereas in the placebo group, “tender” increased by 3% and “tiring-exhausting” had a 4% change (Figure 7).

**Figure 8:**
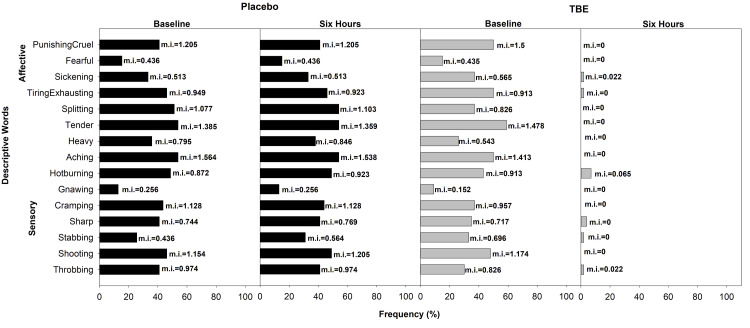
Frequency of subjects describing trunk pain and their corresponding mean pain intensity (mi) using short-form McGill Pain Questionnaire at baseline and at the end of the study after six hours for placebo (n = 39) and turmeric-Boswellia extract (TBE) (n = 46). Mean pain intensity values, shown next to each bar, indicate the mean severity rating assigned by participants. Higher mi values reflect the greater perceived intensity of the described sensation or effect.

In the trunk category of the placebo group, at baseline, 54% reported “aching” and “tender” with mi of 1.56 and 1.39, respectively, in the sensory domain, and 46% reported “tiring-exhausting” in the affective category. Whereas, in the TBE group, 59% reported “tender” (mi=1.48) in the sensory domain, and 50% reported “tiring-exhausting” (mi=0.91) and “punishing-cruel” (mi=1.5) in the affective domain. After the study of 6 h in the TBE group, there was a 100% improvement in “tender” and “punishing-cruel” (Figure 8).

**Figure 9:**
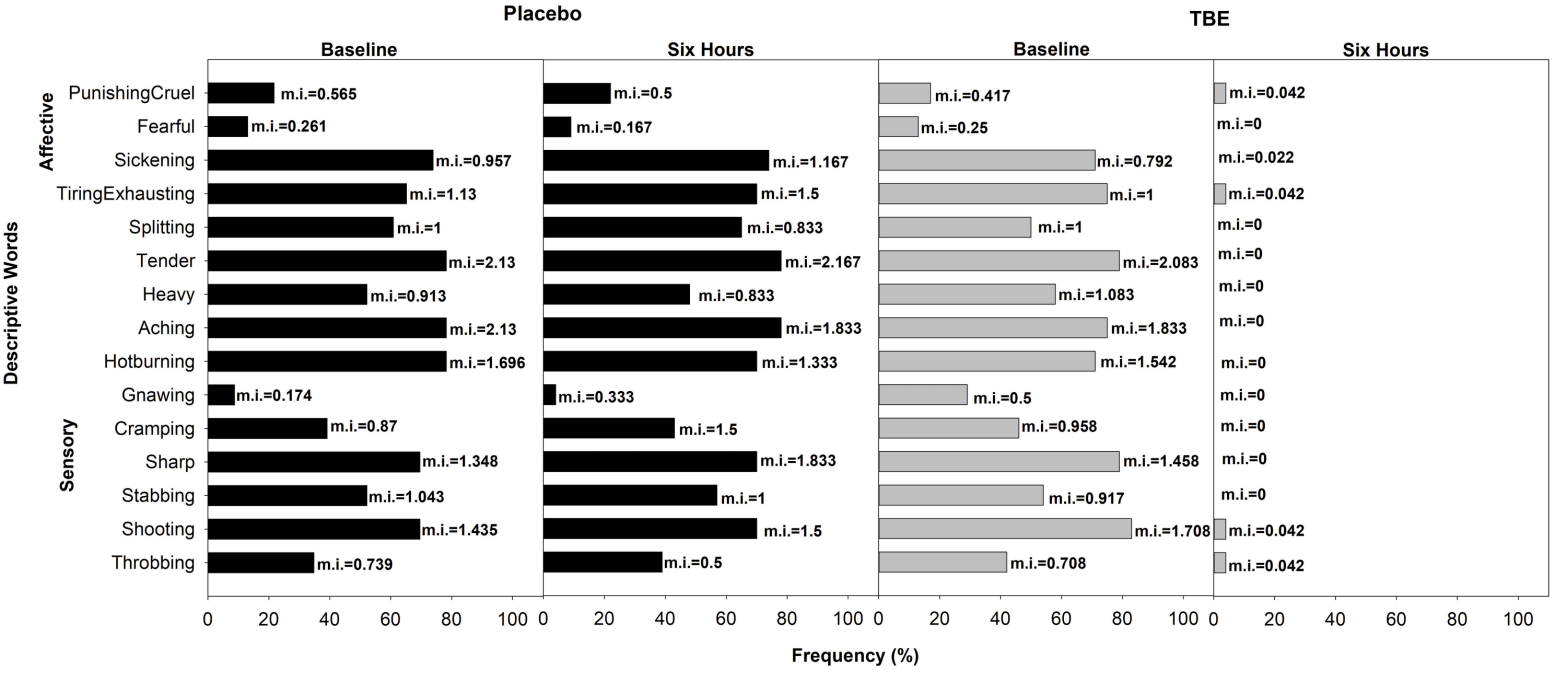
Frequency of subjects describing upper limb pain and their corresponding mean pain intensity (mi) using short-form McGill Pain Questionnaire at baseline and at the end of the study after six hours for placebo (n = 23) and turmeric-Boswellia extract (TBE) (n = 24). Mean pain intensity values, shown next to each bar, indicate the mean severity rating assigned by participants. Higher mi values reflect the greater perceived intensity of the described sensation or effect.

In the upper limbs category, at baseline, 78% reported “hot-burning,” “aching,” and “tender” pain in the sensory domain, and 74% reported “sickening” in the affective domain in the placebo group. In the TBE group, “shooting,” with 83%, was highest in the sensory domain, and “tiring-exhausting,” with 75%, was highest in the affective domain. After the study of six hours in the TBE group, there was a 100% reduction in both sensory and affective domains, while in the placebo group, there was a slight increase in the mi in “aching” and “tender” and a 10% change in “hot-burning” (Figure 9).

The VAS had a significant reduction (p < 0.001) of 97%, and PPI had a significant reduction (p < 0.001) of 96% pain relief in the TBE group compared to the placebo scores of 1% and 2%, respectively (Table 2).

**Table 2:** Comparison of short-form McGill Pain Questionnaire scores (total) before and six hours after administering placebo and TBE group PPI - present pain index; SE - standard error; TBE - turmeric-*Boswellia* extract; VAS - visual analog scale The within-group analysis was conducted using the Wilcoxon signed rank test, and the between-group analysis was conducted using the Mann-Whitney U test.

	Placebo		Within group		TBE		Within group		Between group BL		Between group, six hours	
	Baseline	Six hours	p-value	% change	Baseline	Six hours	p-value	% change	p-value	% change	p-value	% change
	Mean ± SE	Mean ± SE			Mean ± SE	Mean ± SE						
Sensory	11.22 ± 0.54	11.41 ± 0.56	0.0861	-1.69	11.09 ± 0.57	0.18 ± 0.09	<0.001	98.38	0.8069	1.16	<0.001	98.42
Affective	3.01 ± 0.24	2.97 ± 0.23	0.379	1.33	3.10 ± 0.24	0.09 ± 0.05	<0.001	97.1	0.9489	-2.99	<0.001	96.97
Total score	14.23 ± 0.50	14.38 ± 0.53	0.1435	-1.05	14.19 ± 0.54	0.27 ± 0.14	<0.001	98.1	0.8237	0.28	<0.001	98.12
VAS	80.46 ± 1.12	81.48 ± 1.38	0.0012	-1.27	80.67 ± 1.16	2.11 ± 0.79	<0.001	97.38	0.875	-0.26	<0.001	97.41
PPI	4.13 ± 0.07	4.21± 0.08	0.0052	-1.94	4.26 ± 0.06	0.17 ± 0.05	<0.001	96.01	0.1867	-3.15	<0.001	95.96

Anatomical region-wise (general MSP, head and neck, lower limbs, trunk, and upper limbs) descriptives and analysis are given in Table 3.

**Table 3:** Anatomical location of pain-wise analysis of short-form McGill Pain Questionnaire score of placebo and TBE group GMSP - general musculoskeletal pain; TBE - turmeric-*Boswellia* extract Between-group analysis was conducted using the Mann-Whitney U test, where P is the placebo group, and T is the treatment group. N is the total number of participants per group (N = 116 for the treatment group and placebo group). n is the number of participants per group based on the anatomical location of pain.

			GMSP			Head and neck			Lower limbs			Trunk			Upper limbs		
			n	Mean ± SE	p-value (P-T)	n	Mean ± SE	p-value (P-T)	n	Mean ± SE	p-value (P-T)	n	Mean ± SE	p-value (P-T)	n	Mean ± SE	p-value (P-T)
Before administration	Placebo (N = 116)	Sensory	6	12.67 ± 2.65	0.351	8	10.63 ± 1.93	0.124	40	10.65 ± 0.94	0.909	39	10.39 ± 0.92	0.62	23	13.48 ± 1.14	0.923
		Affective	6	3.17 ± 0.31	0.656	8	1.75 ± 0.49	0.291	40	3.2 ± 0.47	0.833	39	3.1 ± 0.45	0.953	23	2.91 ± 0.37	0.306
		Total score MPQ	6	15.83 ± 2.69	0.327	8	12.38 ± 1.64	0.038	40	13.85 ± 0.85	0.888	39	13.49 ± 0.87	0.636	23	16.39 ± 1	0.748
		VAS	6	73 ± 4.28	0.185	8	81.25 ± 5.07	0.832	40	80.15 ± 1.94	0.66	39	83.13 ± 1.93	0.76	23	78.13 ± 2.28	0.655
		PPI	6	3.83 ± 0.48	0.327	8	4.38 ± 0.18	0.334	40	4.2 ± 0.1	0.995	39	4.08 ± 0.11	0.486	23	4.09 ± 0.21	0.28
	TBE (N = 116)	Sensory	4	8.5 ± 3.28	-	6	14.5 ± 0.76	-	36	10.78 ± 0.96	-	46	9.7 ± 0.98	-	24	13.79 ± 1.05	-
		Affective	4	2.75 ± 1.03	-	6	2.5 ± 0.43	-	36	3.28 ± 0.49	-	46	3.41 ± 0.45	-	24	2.46 ± 0.22	-
		Total score MPQ	4	11.25 ± 3.57	-	6	17 ± 0.63	-	36	14.06 ± 0.89	-	46	13.11 ± 0.99	-	24	16.25 ± 0.96	-
		VAS	4	82.5 ± 4.79	-	6	79.67 ± 5.04	-	36	79.03 ± 2.19	-	46	84.04 ± 1.77	-	24	76.63 ± 2.44	-
		PPI	4	4.5 ± 0.29	-	6	4.67 ± 0.21	-	36	4.17 ± 0.12	-	46	4.17 ± 0.1	-	24	4.42 ± 0.15	-
Six hours after administration	Placebo (N = 116)	Sensory	6	13.67 ± 2.88	0.03	8	10.63 ± 1.93	<0.001	40	10.73 ± 0.96	<0.001	39	10.67 ± 0.95	<0.001	23	13.52 ± 1.18	<0.001
		Affective	6	3.33 ± 0.33	<0.001	8	1.75 ± 0.49	0.011	40	3.08 ± 0.44	<0.001	39	3.08 ± 0.45	<0.001	23	2.96 ± 0.37	<0.001
		Total score MPQ	6	17 ± 2.88	0.011	8	12.38 ± 1.64	<0.001	40	13.8 ± 0.92	<0.001	39	13.74 ± 0.9	<0.001	23	16.48 ± 1.05	<0.001
		VAS	6	82.17 ± 4.8	<0.001	8	81.88 ± 5.17	<0.001	40	79.88 ± 2.98	<0.001	39	84.74 ± 2.04	<0.001	23	78.44 ± 2.17	<0.001
		PPI	6	4 ± 0.52	<0.001	8	4.63 ± 0.18	0.001	40	4.2 ± 0.16	<0.001	39	4.21 ± 0.12	<0.001	23	4.13 ± 0.21	<0.001
	TBE (N = 116)	Sensory	4	0 ± 0	-	6	0 ± 0	-	36	0.28 ± 0.25	-	46	0.2 ± 0.1	-	24	0.08 ± 0.08	-
		Affective	4	0 ± 0	-	6	0 ± 0	-	36	0.17 ± 0.17	-	46	0.04 ± 0.03	-	24	0.08 ± 0.06	-
		Total score MPQ	4	0 ± 0	-	6	0 ± 0	-	36	0.44 ± 0.42	-	46	0.24 ± 0.1	-	24	0.17 ± 0.1	-
		VAS	4	0 ± 0	-	6	0 ± 0	-	36	3.19 ± 2.38	-	46	1.96 ± 0.59	-	24	1.67 ± 0.78	-
		PPI	4	0 ± 0	-	6	0 ± 0	-	36	0.19 ± 0.12	-	46	0.2 ± 0.06	-	24	0.17 ± 0.08	-

Mann-Whitney U test shows that at baseline, there was no difference between the groups in sensory and affective domains or total MPQ score, including VAS and PPI score (p > 0.05). At the end of the study, a significant (p < 0.001) reduction in MPQ scores was observed in the TBE group compared to placebo.

## Discussion

Painful stimuli detected by the peripheral nociceptors lie in the sensory domain as pain intensity. The brain processes the sensory information into unpleasant sensations, which lie in the affective domain. The common scales of pain assessment, like VAS and NRS, measure the pain intensity only. The SF-MPQ encompasses both sensory and affective domains and incorporates VAS and a present pain intensity scale. There is evidence that suggests that people judge pain intensity very differently from its unpleasantness whenever separate scales for both domains are given. In certain cases, although peripheral nociceptive transmission can be blocked, pain may yet persist, indicating the affective, motivational aspect of pain.

In an open-label study by Rudrappa et al. on acute MSP comparing turmeric and *Boswellia* extracts in sesame oil (Rhuleave-K) with acetaminophen, both groups were equal in reliving sensory pain, but Rhuleave-K was 8.57 times better than acetaminophen in resolving the unpleasantness and emotional aspects (affective) of pain [12].

In this study, “tiring-exhaustive” and “sickening” were the most frequently used affective pain descriptors, with 55% and 45% in the placebo group and 61% and 53% in the TBE group. The placebo group showed negligible change in the pain condition, often showing a slight increase in the mi of chosen words. TBE showed a 97% and 99% reduction in the mi of “tiring-exhaustive” and “sickening,” with a descriptor frequency reduced to 3% and 1%, respectively. This double-blinded, placebo-controlled multicentric study in 232 subjects validated the finding that turmeric-*Boswellia* formulation in sesame oil has significant action on the affective domain.

Curcumin is known to have numerous targets and mechanisms of action. Anti-inflammatory mechanisms include downregulation of COX-2, lipoxygenase, inducible iNOS enzymes [13,14], and inhibition of tumor necrosis factor-alpha (TNF-α), IL-1, -2, -6, -8, and -12. The transient receptor potential cation channel subfamily V member 1 (TRPV1) plays a role in nociception by providing a sensation of scalding heat and pain. Curcumin has a vanilloid moiety and can competitively block the TRPV1 activation to inhibit TRPV1-mediated pain hypersensitivity [15].

Boswellic acids, mainly AKBA, by inhibiting 5-LOX (5-lipoxygenase), a key enzyme in the leukotriene biosynthesis, reduce leukotrienes in intact neutrophils, which mediate inflammation. It also exhibits anti-inflammatory activity by inhibition of nuclear factor kappa B (NF-κB), TNF-α, IL-6, IL-2, and interferon-gamma (IFN-γ). The investigational product, which has a synergistic blend of *Boswellia* and turmeric extracts in sesame oil, opens up multiple pathways for quick relief of pain.

It is generally thought that different nociceptors and fibers underlie different pain sensations, with the myelinated Aδ fibers responsible for localized “sharp” and “shooting” pain and the unmyelinated C fibers responsible for less localized dull pain sensations [16,17]. The perception of fast pain, also known as sharp pain, occurs very rapidly because the nerve impulses propagate along medium-diameter, myelinated Aδ fibers. Fast pain was not felt in deeper tissues of the body, and pain arising from musculoskeletal tissues was characteristic of deep pain and had important differences from that of cutaneous pain. Widespread pain is commonly seen in MSP conditions, which is characteristic of deep tissue pain that refers to the areas remote from injury. Deep pains are diffuse and difficult to localize and represent a challenge in diagnosis and identification of etiology.

Impulses for slow pain conduct along small-diameter, unmyelinated C fibers. This type of pain, which may be excruciating, is also referred to as aching pain. Slow pain can occur both in the skin and in deeper tissues or internal organs. Cutaneous sensation and pain were usually an indication of impending or actual tissue damage. The sensation of acute muscle pain is the result of Aδ- activation of group-III (fiber) and group-IV (C-fiber) polymodal muscle nociceptors. The nociceptors can be sensitized by the release of neuropeptides from the nerve endings. Aδ fibers are coated with myelin, a fatty substance that enables neurons to transmit impulses very quickly. These fibers are associated with sharp, well-localized, and distinct pain experiences. C fibers transmit impulses more slowly because they are not coated with myelin and seem to be involved in experiences of diffuse dull, burning, or aching pain sensations [18].

In this study, participants used the word “tender” the most, along with “aching” and “hot-burning.” While “tender” and “aching” are of the musculoskeletal classification, “hot-burning” falls in the neuropathic classification. This neuropathic involvement may point to the fact that inflammation as a result of excessive or incorrect exercise activity may be impinging on the nerves. Pain arising from musculoskeletal structures also produces contraction and tenderness in other muscles innervated by the same spinal segment [19,20]. There is some evidence that this spreading muscle contraction plays an important role in clinically significant pains. Participants must, of course, be carefully evaluated to be sure that they do not have undiagnosed underlying disease or injury, and the exclusion criteria in the study eliminated any underlying disease in the group presented with MSP.

The mi score of most descriptors was about one or below one, which means most of them had mild pain except for “aching” and “tender,” which had a score of about 1.7, indicating moderate pain at baseline for both groups. When damage occurs deep within the body, people usually report feeling a “dull,” “aching,” or “throbbing” pain, but damage produced by a brief noxious event to the skin was often described as “sharp” [18,21]. In contrast to sharp, localized characteristics of cutaneous pain, muscle pain was described as aching and cramping with diffuse localization.

In the TBE group, the mi at baseline for “aching” was 1.78, and for “tender,” it was 1.73, which was reduced by 99% for both after six hours post-dose. In the placebo group, the mi was 1.67 and 1.71, which minimally increased to 1.72 and 1.71. The mi for the “throbbing” and “sharp” was (0.65 and 1.02) at baseline in TBE, which was reduced by 97% for both, and the placebo group had negligible change.

Paradoxically, most experimental pain research has been on cutaneous pain, although cutaneous pain was less important than deep tissue pain. In the current study, overall cutaneous pain intensity and frequency of reporting were less than that of MSP intensity in both groups, and it was also true when the mi was considered separately for each location. In the location-wise categorization of MSP, in all five locations, “tiring-exhausting” seems to be the word of choice for the affective domain, followed by “sickening” and “punishing-cruel.” In the sensory domain, “tender” was the choice, followed by “shooting,” “aching,” and “hot-burning.” The pattern found in this study was almost in alignment with the profile of descriptors chosen in the study of MSP by Melzack [6].

This study relies on subjective data provided by the participants without incorporating objective measures such as biomarker analysis, and the absence of a positive control is a limitation. Future research could involve a study population drawn from a global community.

## Conclusions

This study on exercise-induced MSP indicates the fact that the novel formulation of turmeric-*Boswellia* extract in sesame oil reduces the mi of sensory as well as affective pain significantly (p < 0.001) by over 97%. In the location-wise analysis also, a significant (p < 0.001) reduction in the mi of pain descriptive words in general musculoskeletal, head and neck, trunk, lower and upper limbs were seen. Thus, it can be concluded that the turmeric-*Boswellia* formulation was very effective for exercise-induced MSP irrespective of location and has significant pain relief for sensory as well as affective pain sensations.
